# *Pseudocrossidium replicatum* (Taylor) R.H. Zander is a fully desiccation-tolerant moss that expresses an inducible molecular mechanism in response to severe abiotic stress

**DOI:** 10.1007/s11103-021-01167-3

**Published:** 2021-06-29

**Authors:** Selma Ríos-Meléndez, Emmanuel Valadez-Hernández, Claudio Delgadillo, Maria L. Luna-Guevara, Mario A. Martínez-Núñez, Mishael Sánchez-Pérez, José L. Martínez-y-Pérez, Analilia Arroyo-Becerra, Luis Cárdenas, Martha Bibbins-Martínez, Ignacio E. Maldonado-Mendoza, Miguel Angel Villalobos-López

**Affiliations:** 1 Laboratorio de Genómica Funcional y Biotecnología de Plantas, Centro de Investigación en Biotecnología Aplicada, Instituto Politécnico Nacional, C.P. 90700 Tepetitla de Lardizábal, Tlaxcala México; 2 Instituto de Biología, Universidad Nacional Autónoma de México, Ciudad de México, México; 3 Facultad de Ingeniería Química, Benemérita Universidad Autónoma de Puebla, C.P. 72000 Puebla, Puebla México; 4 UMDI-Sisal, Facultad de Ciencias, Universidad Nacional Autónoma de México, C.P. 97302 Mérida, Yucatán México; 5 Unidad de Análisis Bioinformáticos, Centro de Ciencias Genómicas, Universidad Nacional Autónoma de México, C.P. 62210 Cuernavaca, Morelos México; 6 Centro de Investigación en Genética y Ambiente, Universidad Autónoma de Tlaxcala, C.P. 90210 Ixtacuixtla, Tlaxcala México; 7 Instituto de Biotecnología, Universidad Nacional Autónoma de México, C.P. 62210 Cuernavaca, Morelos México; 8 Centro Interdisciplinario de Investigación para el Desarrollo Integral Regional, Unidad Sinaloa, Instituto Politécnico Nacional, C.P. 81049 Guasave, Sinaloa México

**Keywords:** Bryophyte, Moss, Dehydration, Desiccation, Osmotic stress, Salt stress, ABA, Gametophore, Protonema, Transcriptome, RNA-Seq

## Abstract

**Key message:**

The moss *Pseudocrossidium replicatum* is a desiccation-tolerant species that uses an inducible system to withstand severe abiotic stress in both protonemal and gametophore tissues.

**Abstract:**

Desiccation tolerance (DT) is the ability of cells to recover from an air-dried state. Here, the moss *Pseudocrossidium replicatum* was identified as a fully desiccation-tolerant (FDT) species. Its gametophores rapidly lost more than 90% of their water content when exposed to a low-humidity atmosphere [23% relative humidity (RH)], but abscisic acid (ABA) pretreatment diminished the final water loss after equilibrium was reached. *P. replicatum* gametophores maintained good maximum photosystem II (PSII) efficiency (Fv/Fm) for up to two hours during slow dehydration; however, ABA pretreatment induced a faster decrease in the Fv/Fm. ABA also induced a faster recovery of the Fv/Fm after rehydration. Protein synthesis inhibitor treatment before dehydration hampered the recovery of the Fv/Fm when the gametophores were rehydrated after desiccation, suggesting the presence of an inducible protective mechanism that is activated in response to abiotic stress. This observation was also supported by accumulation of soluble sugars in gametophores exposed to ABA or NaCl. Exogenous ABA treatment delayed the germination of *P. replicatum* spores and induced morphological changes in protonemal cells that resembled brachycytes. Transcriptome analyses revealed the presence of an inducible molecular mechanism in *P. replicatum* protonemata that was activated in response to dehydration. This study is the first RNA-Seq study of the protonemal tissues of an FDT moss. Our results suggest that *P. replicatum* is an FDT moss equipped with an inducible molecular response that prepares this species for severe abiotic stress and that ABA plays an important role in this response.

**Supplementary Information:**

The online version contains supplementary material available at 10.1007/s11103-021-01167-3.

## Introduction

Due to climate change, rainfall frequency has decreased, and global temperatures have increased in recent years, resulting in an imbalanced ecosystem and hampering crop productivity (Fedoroff et al. 2010). It is estimated that the world population will reach more than 9 billion by 2050 (Godfray et al. 2010). Studies have suggested that a 70–100 % increase in food production is needed to feed the growing population (Godfray et al. 2010). Among all abiotic factors, drought is the environmental stressor with the highest global agronomic impact (Boyer 1982; Araus et al. 2002; Cramer et al. 2011). In only the past decade, global losses in crop production due to drought have totalled approximately $30 billion (Gupta et al. 2020). Therefore, there is a strong need to understand the mechanisms by which plants adapt to adverse environmental conditions in order to improve stress tolerance.

Drought causes water deficit (Bray 1997) and may even lead to desiccation, a condition in which bound water is the only water that remains in plant cells (Ramanjulu and Bartels, 2002). Although seeds of higher plants can withstand desiccation (Bewley 1979), the vegetative tissues of most plants do not tolerate a water content below 30–60 % (Challabathula and Bartels 2013; Zhang and Bartels 2018). Interestingly, in the course of evolution, some plants have developed desiccation tolerance (DT), which enables them to survive when free water is completely lost from the plant cells. Plants with DT can withstand complete desiccation for long periods and recover normal physiological status rapidly upon rehydration. A small number of vascular plant species have evolved to exhibit vegetative DT; these plants are known as resurrection plants Leprince and Buitink 2015; Giarola and Bartels 2015; Farrant et al. 2015; Zhang and Bartels 2018). Resurrection plants have been successfully explored as sources of genes with biotechnological potential to improve drought tolerance in transgenic plants (Villalobos et al. 2004; Garwe et al. 2006).

Bryophytes were the first plants to colonize terrestrial habitats, an event that required acquisition of unique physiological and molecular mechanisms to withstand fluctuating water availability. The ability to tolerate dehydration is widely distributed among bryophytes, but only a select group shows full DT. Several methods have been proposed to classify DT in mosses (reviewed in Wood 2007). The Austin protocol was proposed to generate a consensus methodology for DT classification in mosses (Wood 2007). This protocol classifies species with DT into two categories: (a) fully desiccation-tolerant (FDT) species, which include moss species that can survive both rapid and slow dehydration and can recover maximum efficiency of photosystem II (PSII) (Fv/Fm) values upon rehydration from a completely desiccated state, and (b) modified desiccation-tolerant (MDT) plants, which include species that survive only slow and moderate water loss (Wood 2007). The moss *Tortula ruralis* has been reported to be an FDT plant that has a constitutive mechanism for survival of desiccation (Oliver et al. 2000). Recently, other mosses, such as *Pterygoneurum lamellatum* (Stark et al. 2013), *Syntrichia caninervis* (Gao et al. 2014), and *Bryum argenteum* (Gao et al. 2015, 2017), have been reported to exhibit DT. *Physcomitrium patens* (formerly named *Physcomitrella patens*) has emerged as a model moss due to several characteristics, such as its ability to grow on simple liquid or solid media, its simple developmental pattern, its responsiveness to common phytohormones, its rapid cell cycle and growth rate, its ability to be transformed at high frequency and its ability to perform double recombination at rates similar to those shown by yeast (Cove 2005; Cove et al. 2006; Rensing et al. 2020). However, *P. patens* has been reported to be a mesic moss (Wood 2007) that cannot survive vegetative desiccation (Frank et al. 2005; Khandelwal et al. 2010; Koster et al. 2010; Komatsu et al. 2013). Additionally, this moss shows an inducible molecular response to dehydration that is mediated, at least in part, by the abiotic stress-related hormone abscisic acid (ABA) (Hiss et al. 2014; Ortiz-Ramírez et al. 2016; Perroud et al. 2018).

Increases in the levels of soluble sugars have been reported to occur under abiotic stress in vascular and nonvascular plants (Bhyan et al. 2012; Dong and Beckles 2019; Mayaba et al. 2001; Minami et al. 2005; Nagao et al. 2006; Cruz de Carvalho et al. 2015). During abiotic stress, soluble sugars play important roles in protecting membranes and macromolecules and in preventing enzymes from denaturing, and soluble sugars accumulate in vacuoles to enable osmotic adjustment (Nagao et al. 2006; Dinakar and Bartels 2013).

Furthermore, a molecular response occurs in response to abiotic stress, and the resulting gene expression tends to allow the plant to adapt to adverse conditions. ABA is a phytohormone that plays important roles in the regulation of many plant responses to different biotic and abiotic stresses and in plant developmental modulation (Finkelstein 2013; Humplík et al. 2017; Ma et al. 2018). The role of ABA in the regulation of the internal mechanisms in response to dehydration and other abiotic factors has been studied in mosses, mainly *P. patens* (Koster et al. 2010; Khandelwal et al. 2010; Takezawa et al. 2011, 2015; Saruhashi et al. 2015). Some core components of the angiosperm ABA-mediated transduction pathway are present in *P. patens*, indicating that some key genes of the pathway are conserved in bryophytes (Komatsu et al. 2020). The transcription factor *ABI3*, originally identified in Arabidopsis by screening for ABA-insensitive mutants (Koornneef et al. 1984), is also present in *P. patens* and plays a crucial role in the acquisition of the ABA-dependent DT of vegetative tissues of this moss (Khandelwal et al. 2010). In *P. patens*, a group A protein phosphatase type 2 C (PP2C), which is related to ABI1, acts as a negative regulator of ABA signalling (Sakata et al. 2009; Komatsu et al. 2009, 2013). Additionally, *PpABI3* is involved in regulating the cold response and freezing tolerance in *P. patens* (Tan et al. 2017). However, bryophytes also express regulatory genes that are not present in vascular plants and are involved in the modulation of the ABA transduction pathway. The *P. patens* gene *ANR* is conserved in algae but absent in vascular plants and encodes a kinase required for DT (Stevenson et al. 2016). *De novo* transcriptome assembly has been performed for gametophores of DT moss species in response to dehydration; the examined species include *S. caninervis* (Gao et al. 2014) and *B. argenteum* (Gao et al. 2015, 2017). However, no studies have analysed the protonemata of a DT moss under conditions of dehydration using RNA-Seq.

Our group is interested in studying the moss *P. replicatum* (Taylor) R. H. Zander, which is widely distributed in the central highlands of Mexico and exhibits an outstanding ability to recover growth after fast desiccation. In the present work, we show that the moss *P. replicatum* is an FDT species harbouring an inducible and rapid molecular mechanism that prepares it for desiccation. This study represents the first RNA-Seq study of protonemal tissues of an FDT moss. We propose *P. replicatum* as a new model for the study of DT in mosses.

## Materials and methods

### Plant material

Samples of *P. replicatum* (Taylor) R. H. Zander were collected during the rainy season in a *Juniperus deppeana* forest in Ixtacuixtla, Tlaxcala, Mexico (19-20-03.3 °N, 98-21-59.9 °W, 2159 m.a.s.l.). For dehydration experiments, hydrated gametophores were collected in the field, maintained in a well-watered state during transportation to the laboratory and immediately used for experimental assays. For microscopy, desiccated samples from the National Herbarium of Mexico (MEXU) were used. A distribution map was generated using data from the MEXU records (http://www.ib.unam.mx/botanica/herbario/).

### DT assay

The level of DT of *P. replicatum* was investigated according to the Austin protocol (Wood 2007). Briefly, freshly collected gametophores were carefully washed, and hydrated tissue samples (24 h at 90% relative humidity (RH), 20 °C) were equilibrated at two RH regimes (23% RH for fast dehydration and 67% RH for slow dehydration) for 1 or 7 d. As specified in the Austin protocol, we used saturated NH_4_NO_3_ and KCH_3_CO_2_ solutions to obtain 67% RH (− 54 MPa) and 23% RH (− 198 MPa), respectively. During dehydration, the maximum quantum yield (QY) of PSII was measured using a fluorometer (Fluorpen FP 100, PSI Instruments, Czech Republic). In dark-adapted samples, the QY is equivalent to the Fv/Fm. The samples were collected after the indicated durations of rehydration with distilled water, and the QY was measured in dark-adapted samples (10 min).

### Effects of ABA on the dehydration kinetics and Fv/Fm values of *P. replicatum* gametophores

Freshly collected gametophores were carefully washed, and groups of hydrated samples (100 mg) were incubated in 50 ml conical Falcon tubes containing 5 ml of distilled water. Some samples were treated with ABA (10 or 100 µM) and incubated for 60 min with gentle shaking. Furthermore, samples were subjected to fast or slow dehydration by exposure to 23 or 67 % RH, respectively, as previously described. Before dehydration, the excess water was carefully removed with blotting paper. Water loss was calculated on a fresh-matter basis by recording the fresh weights of the samples until no further change was noted (Koster et al. 2010). The Fv/Fm values of the samples were recorded during the dehydration process.

### Effects of protein synthesis or transcription inhibitors on the DT of *P. replicatum* gametophores

Essentially, a modified version of the Austin protocol was used. Gametophores were incubated with protein synthesis inhibitors or a transcription inhibitor. The inhibitors were applied before or after dehydration, and the recovery of the Fv/Fm was measured in rehydrated tissues. For application of inhibitors before dehydration, we used freshly collected gametophores that were maintained in a fully hydrated state at the laboratory. Individual groups of samples were pretreated for 24 h with different combinations of the translation inhibitors cycloheximide (300 µM, CHX), chloramphenicol (3 mM, CMP) (Proctor et al. 2007; Proctor and Smirnoff 2000), and α-amanitin (10 µM) (Rajjou et al. 2004) with or without ABA (10 µM) and further equilibrated to 67% RH for 24 h. Rehydrated samples were used to measure the Fv/Fm at 1 and 24 h. For application of inhibitors after dehydration, fully hydrated gametophores were equilibrated to 67% RH for 24 h. Furthermore, the dehydrated samples were rehydrated for 24 h with water to which different combinations of CHX, CMP, α-amanitin, and ABA had been added at the described concentrations. Finally, the PSII efficiency was measured at the indicated times.

### Quantitative determination of total soluble sugars

For each treatment, 100 mg of freshly collected fully hydrated gametophores were incubated for 24 h in PpNH_4_ liquid medium supplemented with 400 mM sorbitol, 200 mM NaCl, or 10 µM ABA in an environmentally controlled room (photoperiod 16/8 h light/dark, 24 °C) with gentle shaking (100 rpm). The medium was discarded, and the excess liquid was removed carefully using absorbent paper. The samples were transferred to liquid nitrogen, pulverized, and homogenized at 0 °C in 5 ml of EtOH-H_2_O (4:1). The homogenate was centrifuged at 14,000×*g* for 5 min at 4 °C, and the supernatant was recovered. After evaporation of the ethanol, the samples were dissolved in distilled H_2_O (0.5 ml) and centrifuged at 14,000×*g* for 5 min at 4 °C to remove any debris. The soluble sugars in the supernatant were quantified with an anthrone-sulfuric acid assay using glucose as a standard (Yemm and Willis 1954; Komatsu et al. 2013).

### Spore germination analysis and *in vitro* culture of *P. replicatum*

Axenic *in vitro* germination of *P. replicatum* spores was performed by superficial sterilization of *P. replicatum* sporophytes with sodium dichloroisocyanurate at 0.02% for 30 min followed by at least three washes with sterile water. Spores were released in 1 ml of sterile water with the aid of a needle, and serial dilutions were plated on PpNH_4_ solid medium. The germination of spores was scored every 24 h using a Nikon SMZ1500 stereomicroscope. Emerging protonemal cells with at least half the diameter of the spore were considered fully germinated. To obtain a monosporic pure line of *P. replicatum*, the resulting protonema from one individual spore was selected and cultured in PpNH_4_ medium under standard growth conditions in a growth room at 24 °C under a 16/8 h light/dark photoperiod with a light intensity of 55 µmol photons m^− 2^ s^− 1^. For analysis of the effect of ABA on protonema development, individual protonemal filaments were followed for 7 d using a Nikon TE300 inverted microscope with a 10× water immersion lens (Nikon).

### RNA extraction for transcriptome analysis

For analysis of transcriptomes, total RNA was extracted with TRIzol reagent (Thermo Fisher Scientific, USA) from 7-d-old monosporic protonemal tissue, which was dehydrated by exposure to 23% RH (− 198 MPa) for 10 min (T1) or 30 min (T2). RNA from nondehydrated samples was extracted and used as a control (C). Equal amounts of total RNA obtained from three independent biological replicates were pooled for each treatment.

### RNA-Seq library construction and sequencing

To compare the *P. replicatum* transcriptome under control versus dehydration conditions (T1 and T2), libraries were constructed for the different treatments using a TruSeq RNA Sample Preparation Kit following the manufacturer’s instructions (Illumina, Inc., San Diego, CA, USA), and single-end sequencing of the resulting libraries was carried out using an Illumina Genome Analyzer II. Briefly, poly(A)-tailed mRNA was enriched, fragmented, and used for first-strand cDNA synthesis. Subsequently, the second of strand cDNA was synthesized, and the final reactions were cleaned up before performing the end repair step. Furthermore, a single ‘A’ base was added to the 3´ ends of each fragment. Adapters were ligated to both ends of the short fragments, which were enriched by PCR to create the final double-stranded cDNA libraries. Finally, library quality control and quantification were performed with a Bioanalyzer Chip DNA 1000 series II (Agilent) and sequenced directly using a high-throughput Illumina HiSeq sequencing system (Illumina, Inc., San Diego, CA, USA). The raw reads for each library were deposited in the NCBI database, and the data can be downloaded from the Sequence Read Archive (SRA) under BioProject accession number PRJNA601777.

### Global gene expression and differential gene expression analysis

To analyse the RNA-Seq data of *P. replicatum*, Trinity-v2.9.1 https://github.com/trinityrnaseq/trinityrnaseq/wiki programs were used for RNA-Seq *de novo* analysis, and the reference genome of *P. patens* (v3.3) was downloaded from the Phytozome database (Lang et al. 2018). First, quality analysis was performed with the program FastQC v0.11.8 (Andrews 2010), and the adapters were removed from the obtained reads with Trim Galore v0.6.6 with the default parameters (https://www.bioinformatics.babraham.ac.uk/projects/trim_galore/). Next, the reads were assembled using Trinity pipeline programs, and annotation was performed with Trinotate v3.1.1 (Bryant et al. 2017) and eggNOT v5.0 (Huerta-Cepas et al. 2019). Gene expression was quantified and evaluated using the Kallisto program (Bray et al. 2016). Genes with changes in their expression levels were selected via statistical testing with edgeR (Robinson et al. 2010), and the identified transcripts with reported counts were BLASTed with the reference genome of *P. patens.*

## Results

### *P. replicatum* is a moss widely distributed in Mexico

**Fig. 1:**
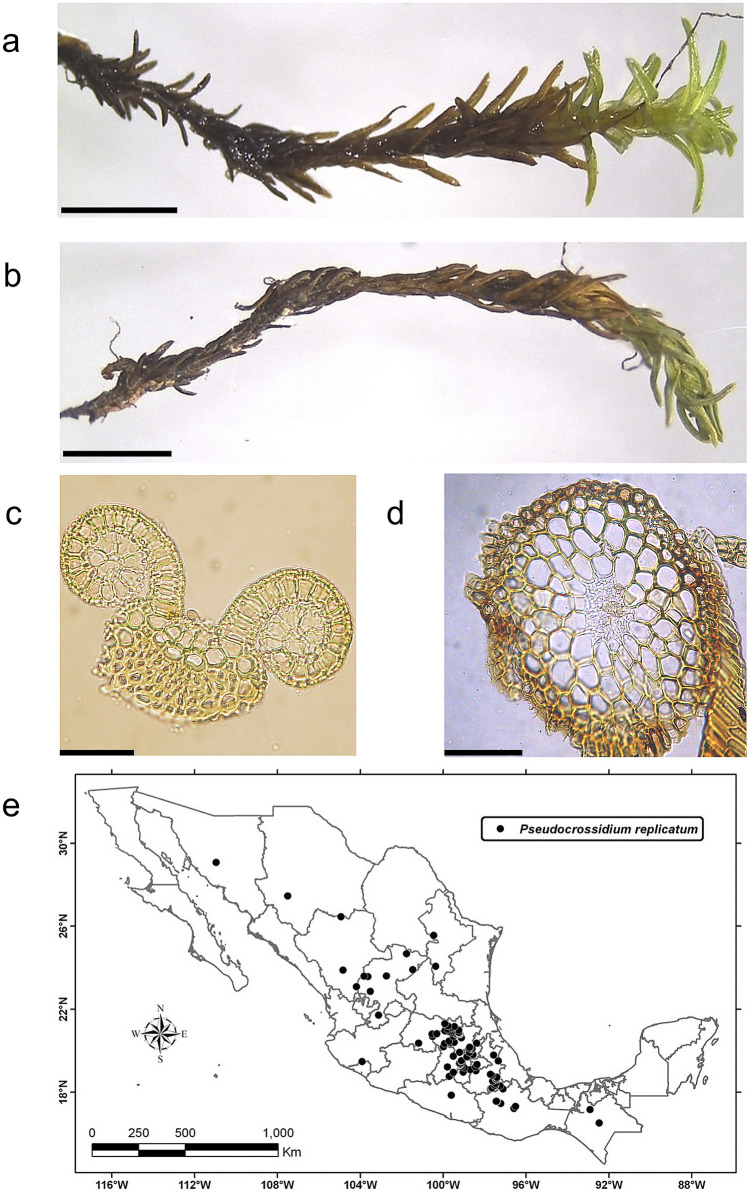
The species ***P. replicatum*** (Taylor) R. H. Zander is widely distributed in Mexico. The pictures show the appearance of *P. replicatum* gametophores when hydrated (**a**) or dehydrated (**b**). Transverse hand-cut sections of leaves (**c**) and stems (**d**) are also shown. According to the registers of MEXU, *P. replicatum* is widely distributed across Mexico (**e**) but is especially enriched in the central highland regions of the country

In the present work, our first aim was to obtain plant material with putative DT characteristics, specifically in the central highlands of Mexico. We identified several moss and other species with interesting characteristics; however, *P. replicatum* stood out and was selected for thorough characterization (Fig. 1a). *P. replicatum* is a moss belonging to the Pottiaceae family that grows on soil or rocks of calcareous origin and has green stems ranging from 3 to 15 mm in height with a central strand (Fig. 1d). The stem leaves are elliptic to ovate-lanceolate and 1–2 mm long with spirally revolute margins (Fig. 1c). The leaves are spirally appressed when dry (Fig. 1b). Interestingly, the photosynthetic system in *P. replicatum* leaves is represented by thin-walled cells in the spiral revolute leaf margins. This moss is widely distributed in Mexico (Fig. 1e) and can be found at different elevations ranging from 415 to 3600 m.a.s.l.

### *P. replicatum* is an FDT moss

**Fig. 2:**
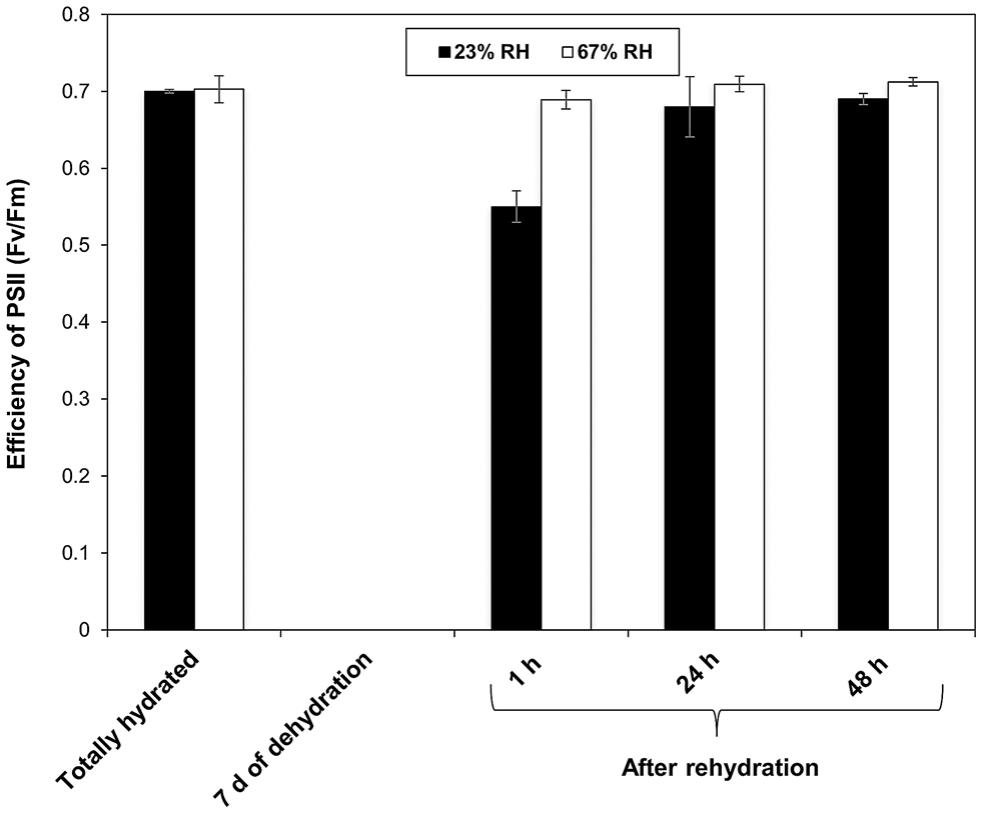
*P. replicatum* is an FDT moss. *P. replicatum* is an FDT moss that can recover its Fv/Fm to normal levels rapidly after prolonged exposure to 23% RH (black bars) or 67% RH (white bars). Totally hydrated gametophores prior to dehydration are indicated as controls for both groups of samples. The data shown are the means ± SDs from three independent experiments, with n = 5 at each indicated time

The Austin protocol (Wood 2007) was used to determine the level of DT exhibited by freshly collected gametophores. The gametophores were equilibrated for 7 d to water potentials of − 54 MPa (67% RH) and − 98 MPa (23% RH), and the Fv/Fm was undetectable under both conditions. Furthermore, the samples were watered, and the Fv/Fm values were evaluated after 1, 24 and 48 h of rehydration. Interestingly, despite such stress conditions, the Fv/Fm values were restored to values corresponding to 95% of the control levels after only 1 h of rehydration and showed Fv/Fm values higher than 0.7 only 2 h after rehydration (Fig. 2). Thus, the results clearly indicated that *P. replicatum* is an FDT moss that rapidly can recover its Fv/Fm to normal levels after prolonged exposure to 67% RH or 23  RH.

### Effects of exogenous ABA on the dehydration and rehydration kinetics and Fv/Fm of *P. replicatum* gametophores

**Fig. 3:**
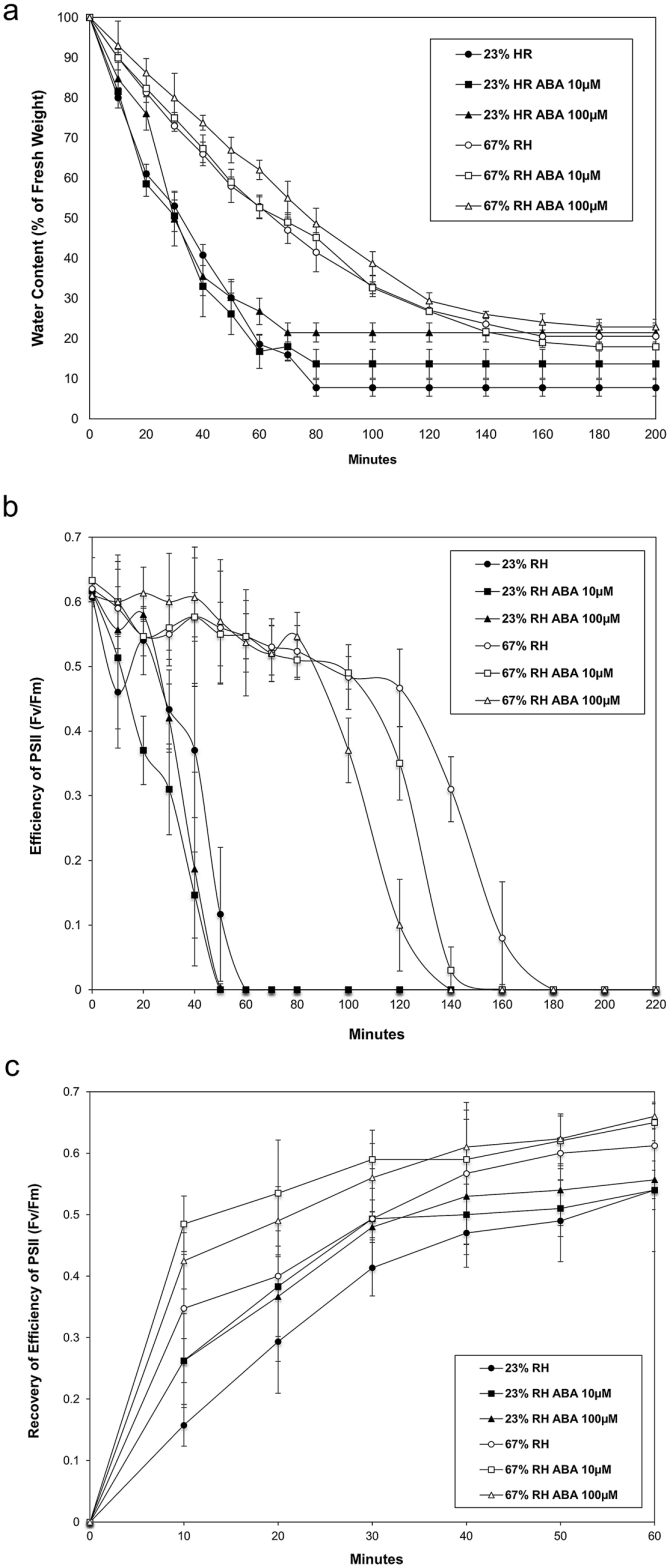
Effects of ABA on dehydration kinetics and the Fv/Fm during the dehydration/rehydration process of gametophores of *P. replicatum*. **a** *P. replicatum* gametophores lost their water content when exposed to a low-humidity atmosphere (23% RH, solid symbols), but ABA pretreatment significantly diminished the final water loss after equilibrium was reached at this humidity level (ANOVA, p value = 0.05). The results obtained when gametophores were exposed to 67% RH are shown as open symbols. **b** *P. replicatum* gametophores maintained high Fv/Fm values for up to two hours during a slow dehydration process; however, ABA pretreatment induced a decrease in the Fv/Fm. **c** ABA pretreatment before dehydration resulted in a faster recovery of the Fv/Fm during the first 20 min of the rehydration process. Each point on the graph represents the average from three independent experiments, each with n = 5

In *P. patens* and other mosses, ABA induces an increase in tolerance to diverse abiotic factors (Werner et al. 1991; Beckett et al. 2000; Beckett 2001; Minami et al. 2005; Nagao et al. 2005; Frank et al. 2005; Khandelwal et al. 2010; Koster et al. 2010; Komatsu et al. 2013). To study the effect of ABA on the dehydration kinetics of *P. replicatum*, freshly collected gametophores were subjected to fast or slow dehydration by exposure to 23% (− 198 MPa) or 67% RH (− 54 MPa), respectively, and the results were analysed using ANOVA. *P. replicatum* gametophores lost approximately 50% of their water content when exposed to a low-humidity atmosphere for only 30 min; however, at 67% RH, an exposure duration greater than 70 min was required to reach a similar percentage of water loss. ABA pretreatment slowed this dehydration process at both 23 RH and 67% RH (Fig. 3a). Under 23% RH conditions, the samples reached equilibrium after 80 min of dehydration; in contrast, the samples reached equilibrium after 180 min of dehydration under 67% RH. Additionally, we analysed the effect of ABA on the Fv/Fm values of *P. replicatum* gametophores exposed to dehydration under 23% RH and 67% RH atmospheres. ANOVA determined that a significantly different Fv/Fm response was observed between the two dehydration treatments (Fig. 3b). The Fv/Fm decayed rapidly at 23% RH, showing a drastic reduction after 30 min of treatment, but 60 min of treatment was required for the Fv/Fm values to become undetectable. Lower Fv/Fm values were recorded under the same conditions when the gametophores were pretreated with ABA after 40 min of dehydration. On the other hand, high oscillatory values of Fv/Fm were obtained under slow-dehydration conditions, even after 80 min of dehydration; ultimately, more than 180 min of dehydration was required for the Fv/Fm values to become undetectable. In contrast, when gametophores pretreated with 100 µM ABA were subjected to slow dehydration, the Fv/Fm became undetectable in 140 min. For both dehydration conditions analysed, we observed faster recovery of Fv/Fm values during the first 20 min of rehydration when ABA pretreatment was applied to the gametophores before dehydration than when ABA pretreatment was not applied (Fig. 3c). Notably, the Fv/Fm values of *P. replicatum* gametophores that were previously exposed to − 54 MPa or − 198 MPa were able to recover to 52 and 80 % of the control Fv/Fm values, respectively, after only 10 min of rehydration. These data suggest that ABA plays a role in the responses of PSII during the dehydration of *P. replicatum*.

### Analysis of the effects of inhibitors of transcription and translation on the recovery of PSII efficiency after dehydration

**Fig. 4:**
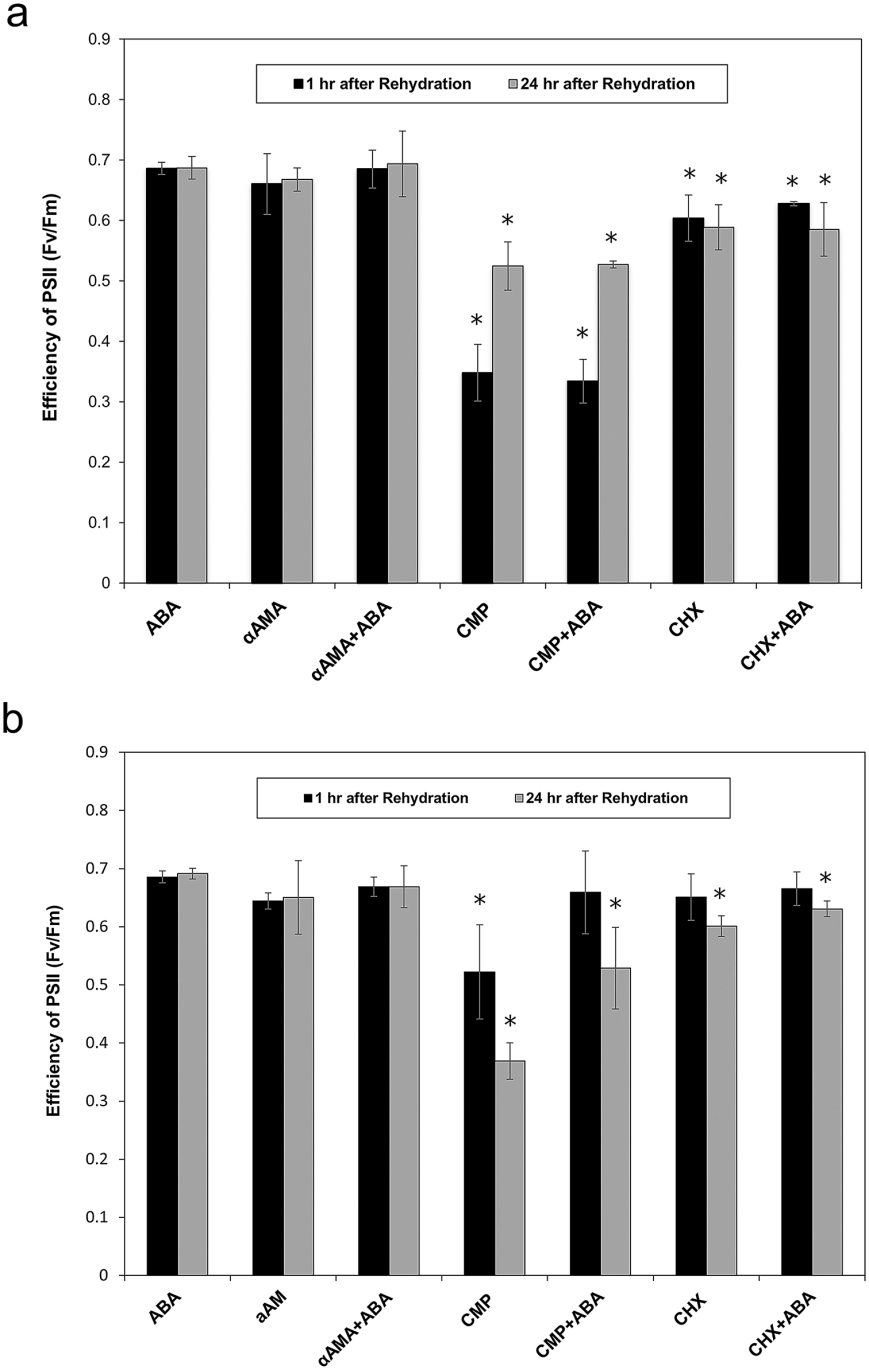
Inhibitor treatment reveals the participation of chloroplastic proteins required for the optimal function of PSII after dehydration. **a** Fv/Fm values obtained when inhibitors were applied before dehydration. **b** Fv/Fm responses obtained when inhibitors were applied during rehydration. Lower values of Fv/Fm were observed when CMP was applied before dehydration than when inhibitors were applied during rehydration. Each point on the graph represents the average ± SD from three independent experiments, each with n = 5. Samples showing significant differences according to ANOVA are indicated by asterisks (p value = 0.05)

To investigate whether the gametophores of *P. replicatum* require the expression of genes induced during dehydration to recover photosynthesis upon rehydration, a modified Austin protocol was conducted. α-Amanitin was used as an inhibitor of transcription, whereas CHX and CMP were used as inhibitors of protein synthesis. The inhibitors were applied independently before dehydration or during rehydration (Fig. 4), and the Fv/Fm values were measured after 1 and 24 h. The Fv/Fm values were analysed using ANOVA. Interestingly, an important delay in Fv/Fm recovery was observed in response to translational inhibitor treatment. Lower values of Fv/Fm were observed when CMP was applied before dehydration (Fig. 4a) than when inhibitors were applied during rehydration (Fig. 4b). Additionally, application of exogenous ABA together with the inhibitors was not enough to restore the Fv/Fm recovery timing, indicating that the DT exhibited by *P. replicatum* gametophores requires *de novo* protein synthesis, which could help to protect the photosynthetic apparatus from damage provoked by stress. α-Amanitin was not observed to have a clear effect under the tested conditions.

### Effect of abiotic stress on the accumulation of soluble sugars in *P. replicatum* gametophores

**Fig. 5:**
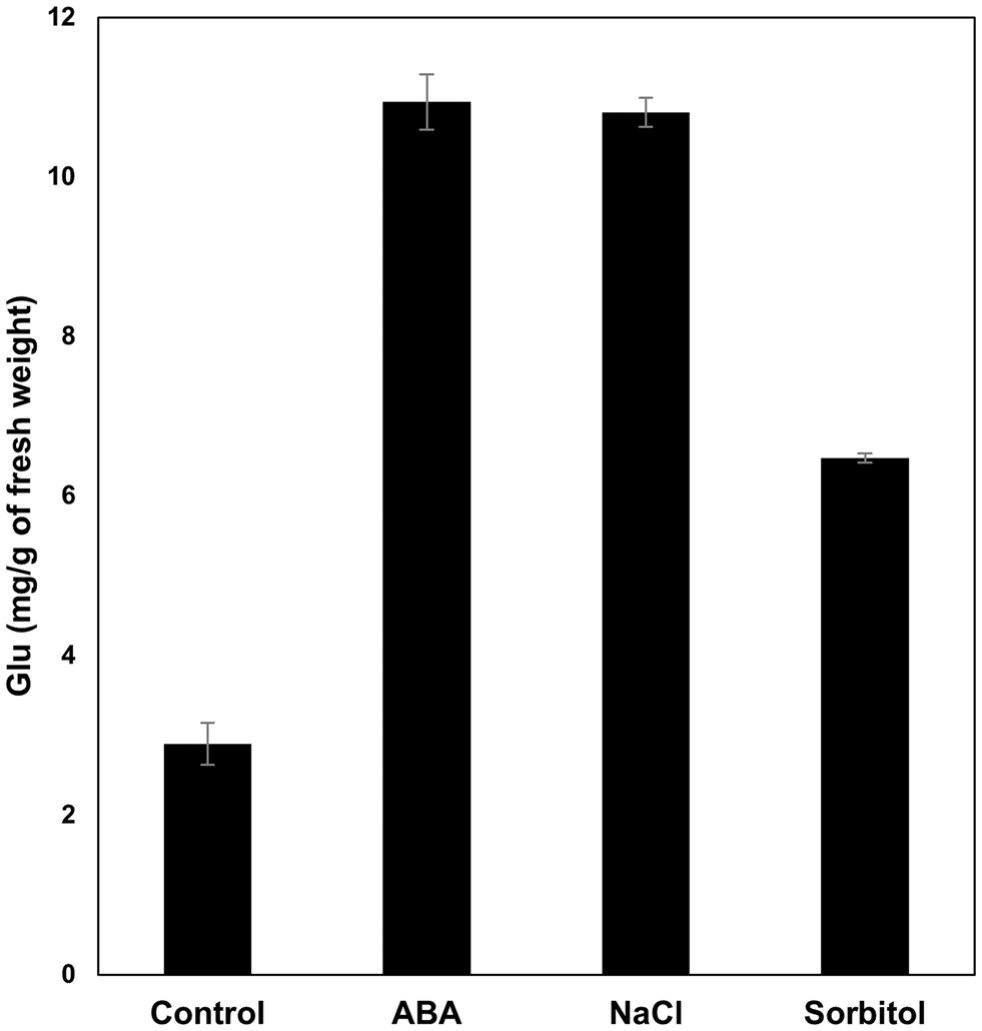
The gametophores of *P. replicatum* accumulate soluble sugars in response to ABA and abiotic stress factors. For each treatment, 100 mg of freshly collected fully hydrated gametophores were incubated for 24 h in PpNH_4_ liquid medium supplemented with 400 mM sorbitol, 200 mM NaCl, or 10 µM ABA. The soluble sugars were quantified with an anthrone-sulfuric acid assay. The bars represent the averages ± SDs from three independent experiments

During abiotic stress, soluble sugars play important roles in protecting membranes and macromolecules and in preventing enzymes from denaturing, and soluble sugars accumulate in vacuoles to enable osmotic adjustment (Nagao et al. 2006; Dinakar and Bartels 2013). Experiments were conducted to determine whether the accumulation of soluble sugars is part of the strategy of the gametophores of *P. replicatum* to withstand abiotic stress. Our results indicated that application of 10 µM ABA or 200 mM NaCl induced strong accumulation of soluble sugars, resulting in levels nearly four times higher than control levels (Fig. 5). On the other hand, a twofold increase in soluble sugars was observed when gametophores were treated with 400 mM sorbitol. These observations indicate that ABA stimulation and NaCl stimulation are sufficient to induce accumulation of soluble sugars in *P. replicatum* gametophores and that moderate osmotic stress also induces sugar accumulation.

### Effects of exogenous ABA on spore germination and protonemal development

**Fig. 6:**
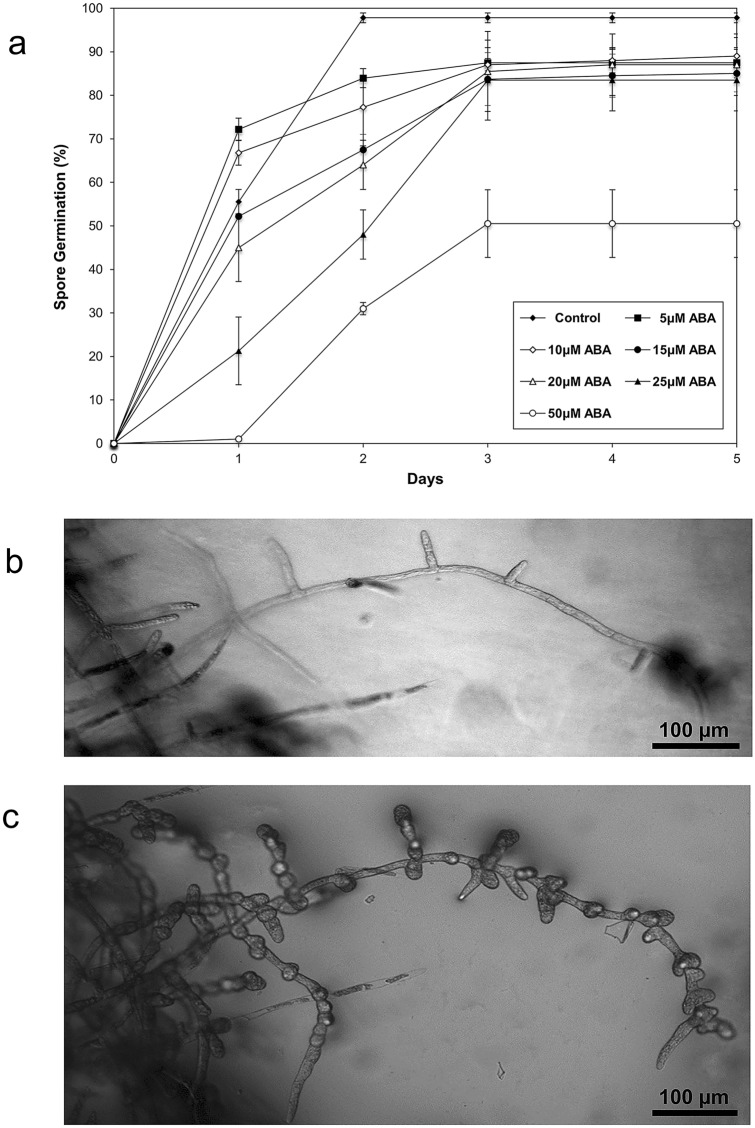
Effects of exogenous ABA on *P. replicatum* spore germination and protonemal development. **a** Each point on the graph represents the average ± SD from three independent experiments, each with n = 120–150. Additionally, an individual protonemal filament from a monosporic line was used to analyse the influence of ABA (10 µM) on protonemal development, where **b** is t0 and **c** corresponds to 7 d of exposure

ABA is a phytohormone that also plays a role in spore germination and protonemal development (Vesty et al. 2016; Decker et al. 2006; Takezawa et al. 2011; Stevenson et al. 2016; Shinozawa et al. 2019; Arif et al. 2019). A putative effect of exogenous ABA on spore rate germination in *P. replicatum* was investigated. Compared with the control, all ABA concentrations tested (5–50 µM) delayed the germination kinetics (Fig. 6a). However, the highest concentration used (50 µM) not only delayed germination but also diminished the germination rate to approximately 50 %. The effect of exogenous ABA on protonemal development was also investigated by observing individual protonemal filaments for 7 d under a microscope. Application of ABA induced clear morphological changes in the protonemal cells of *P. replicatum* (Fig. 6b and c). While the apical cells maintained polarized growth in response to ABA, the nonapical cells of protonemal filaments acquired a small and rounded phenotype with suppressed cell expansion in response to ABA, resembling brachycytes (brood cells).

### RNA-Seq analysis of protonemata in response to dehydration

To investigate the potential existence of a dehydration-inducible system at the transcriptional level, a preliminary analysis of the transcriptome changes of *P. replicatum* in response to dehydration was conducted. The transcriptomes of protonemal samples dehydrated at 23% RH (− 198 MPa) for 10 or 30 min were compared with those of fully hydrated samples. These specific dehydration times were chosen because analysis of the dehydration kinetics of *P. replicatum* protonemata exposed to a 23 % RH atmosphere indicated that 10 and 30 min of treatment caused water content decreases of 30 and 70%, respectively (Supplementary file 1a). A total of 1368 genes were identified to exhibit expression changes in protonemal samples dehydrated for 10 min (T1). However, the number of genes with expression changes increased to 4220 (T2) when dehydration was extended to 30 min. A total of 1973 differentially expressed genes (DEGs) were identified using statistical analysis (Supplementary file 3); 972 corresponded to upregulated transcripts, and 821 corresponded to downregulated transcripts. When the upregulated and downregulated genes for all treatments (T1 + T2) were considered together, annotation analysis identified 175 DEGs with homology to *P. patens* sequences (Supplementary file 1b-A). Only 9 DEGs were detected in T1 in comparison with the hydrated control (C) (Supplementary file 1b-A). However, the number of DEGs increased to 118 when the dehydration treatment was prolonged to 30 min (Supplementary file 1b-A). Interestingly, 137 DEGs were found in T2 compared to T1, 86 of which were also DEGs in T2 compared with C (Supplementary file 1b-A). In total, 154 upregulated genes were identified in T1 + T2 compared with C, and the treatment with the most abundant upregulated genes was T2 (Supplementary file 1b-B). In contrast, a total of 27 genes were downregulated in T1 + T2 in comparison with C (Supplementary file 1b-C), and 21 of these genes were downregulated specifically in T2 (Supplementary file 1b-C). An analysis of only the nonredundant DEGs that were related to *P. patens* identified a total of 89 DEGs, 30 of which were shared between T1 and T2 (Supplementary file 1b-D; Supplementary file 2). A complete list of DEGs is provided in Supplementary file 3. To further analyse the obtained data, the 2-kb regions corresponding to the promoters of all DEGs were analysed using the Plant Cis-Acting Regulatory Elements (PlantCARE) database (Lescot et al. 2002), and we observed the presence of several ABRE and DRE motifs in the promoters of ABA- and drought-responsive genes (Supplementary file 1c). Furthermore, a Gene Ontology (GO) enrichment analysis was performed to compare and plot the induced and repressed genes based on a GO standardized directed acyclic graph (DAG) structured vocabulary system (Supplementary file 1d). Prediction of the subcellular localization of the products of the induced and repressed genes using CELLO2GO (Yu et al. 2014) revealed that chloroplastic protein-encoding genes were the most abundant group among the induced genes (28 %); in contrast, nuclear protein-encoding genes were the most abundant group among the repressed genes (31.8 %) (Supplementary file 1e-A and Supplementary file 1e-B, respectively).

## Discussion

### Anatomical and ecological characteristics of *P. replicatum*

Among plants collected from the central highlands of Mexico, we identified the moss *P. replicatum*, which stood out as an FDT plant species and was selected for further characterization. The Mexican central highlands are surrounded by three volcanoes, one of which is still active (Popocatepetl). In Mexico, this plant species has a broad distribution up to 3,200 m.a.s.l. (Fig. 1e) but is enriched in desert areas and forests in high, mountainous regions with cold weather. In the region of sample collection, the average annual minimum and maximum temperatures are 1.5 and 25 °C, respectively. The estimated annual precipitation is 700 mm, concentrated mainly from June to September, and the dry season spans several months. The leaves of *P. replicatum* adopt a spiral arrangement around the stem during the dehydration process, becoming tightly entwined when the desiccated state is reached. The reason for the evolution of this response is not known, but we suggest that this phenomenon may help *P. replicatum* maintain its internal water content by diminishing the rate of water loss during dehydration. Recently, the chloroplastic and mitochondrial genomes of *P. replicatum* were reported Cevallos et al. 2019, 2020, respectively). A phylogenetic analysis using 16 chloroplast protein-coding genes demonstrated that *P. replicatum* is a member of the Pottiaceae family and is closely related to *Syntrichia ruralis* (Cevallos et al. 2019).

### *P. replicatum* is an FDT moss

We classified *P. replicatum* as an FDT moss. After plants were equilibrated for 7 d to water potentials of − 54 MPa (67% RH) and − 198 MPa (23% RH), the Fv/Fm values of the gametophores was undetectable. However, gametophores of *P. replicatum* recovered normal Fv/Fm levels after rehydration regardless of whether they were desiccated at − 54 MPa or at − 198 MPa, although a faster recovery was observed for the slower dehydration treatment. This observation might suggest that to fully recover the Fv/Fm, *P. replicatum* gametophores must activate an inducible molecular mechanism to quickly repair the damage generated during desiccation. *T. ruralis*, the most studied DT moss, has been demonstrated to employ a constitutive mechanism that helps it withstand desiccation (reviewed in Oliver and Bewley 1997; Oliver and Wood 1997; Oliver et al. 1997; Wood et al. 2000). However, *T. ruralis* also harbours an inducible molecular mechanism that is activated only in the rehydration stage and is dedicated to repair of membrane damage, fragmentation of vacuoles and chloroplasts, depolymerization of microtubules, and denaturation of proteins, among other alterations (Oliver and Bewley 1997; Oliver and Wood 1997; Pressel and Duckett 2010). Other mosses seem to have different strategies to withstand abiotic stress. Although *P. patens* is not considered to be a DT moss with a constitutive mechanism of protection from desiccation (Pressel and Duckett 2010; Koster et al. 2010), it has been shown that this moss has an inducible molecular mechanism that is activated in response to cold (Beike et al. 2015), dehydration (Koster et al. 2010), and ABA and salt treatments (Khraiwesh et al. 2015). Recently, it has been reported that 6 and 9 d of acclimation to 69 % RH are required to induce DT at 33 % RH in protonemal and gametophore tissues of *P. patens*, respectively (Rathnayake et al. 2019). The results obtained using the Austin protocol indicate that *P. replicatum* is an FDT moss that is able to recover photosynthesis after rapid and slow dehydration.

### ABA plays roles in the dehydration process and in the efficiency of PSII in *P. replicatum*

To gain insight into the possible existence of an inducible molecular mechanism in *P. replicatum* that is activated in response to abiotic stress, we first investigated whether ABA plays a role during dehydration in *P. replicatum*. Our results indicate that ABA plays a crucial role in *P. replicatum* responses during dehydration of gametophores. Water loss was slightly reduced when *P. replicatum* gametophores were pretreated with ABA before dehydration (Fig. 3a). Similar responses have been reported for *P. patens* (Koster et al. 2010); ABA pretreatment also increases water retention in this moss during drying at − 52 MPa (68% RH). In contrast, the dehydration kinetics of the moss *Funaria hygrometrica* are not significantly altered by pretreatment with ABA (Werner et al. 1991). However, in other bryophytes such as the liverwort *Marchantia polymorpha*, ABA also decreases the rate of water loss in gametophytes under dehydration (Pence et al. 2005). More studies are needed to understand the different effects of ABA on the dehydration kinetics of bryophyte tissues at several developmental stages. The effect of ABA on photosynthesis was also evaluated in the current study (Fig. 3b). Application of exogenous ABA induced a strong decrease in PSII activity, which was evident when gametophores were subjected to slow dehydration (67% RH). Under controlled conditions, during both rapid and slow dehydration, the QY values became undetectable only after the water content had decreased to approximately 20%. This water content value was reached in 60 and 180 min under rapid and slow dehydration, respectively. However, when ABA pretreatment was applied, a decrease in QY was observed, especially under the slow dehydration treatment. This observation is in accordance with the responses reported for the mosses *Atrichum undulatum* and *Atrichum androgynum*, in which ABA pretreatment reduces several photosynthetic parameters (Beckett 2001; Mayaba et al. 2001). In diverse plant models, such as Arabidopsis and Craterostigma, it has been established that ABA participates in the induction of dehydration and DT (Finkelstein 2013; Sah et al. 2016; Vishwakarma et al. 2017). In bryophytes such as *P. patens* and *F. hygrometrica*, the role of ABA in acquisition of tolerance to abiotic stress has been demonstrated by evidence such as visible cellular phenotypes, plasmolysis, and growth (Werner et al. 1991; Minami et al. 2005; Khandelwal et al. 2010; Bhyan et al. 2012; Stevenson et al. 2016). Interestingly, in the present study, *P. replicatum* gametophores required only 1 h of pretreatment with ABA to show accelerated Fv/Fm recovery upon rehydration of desiccated gametophores, which occurred at least during the first 20 min (Fig. 3c); these findings demonstrate the role of ABA in the rapid photoprotection and/or repair of PSII in mosses during rehydration. Moreover, the results obtained using inhibitors of transcription and translation suggest that the *de novo* expression of chloroplastic proteins is very important for the protection and repair of PSII during the *P. replicatum* dehydration/rehydration response (Fig. 4). Altogether, these observations suggest that ABA induces a molecular response in *P. replicatum* to rapidly prepare this moss to withstand desiccation stress. Thus, once equipped with dehydration- or ABA-induced expression of a set of protective or reparative enzymes, this moss can readily and efficiently respond to dehydration stress.

### ABA plays a role in the accumulation of soluble sugars in *P. replicatum* in response to abiotic stress

Increases in soluble sugars have been reported to occur under abiotic stress in vascular and nonvascular plants (Bhyan et al. 2012; Dong and Beckles 2019; Mayaba et al. 2001; Minami et al. 2005; Nagao et al. 2006; Cruz de Carvalho et al. 2015). During abiotic stress, soluble sugars play important roles in protecting membranes and macromolecules and in preventing enzymes from denaturing, and they accumulate in vacuoles for osmotic adjustment (Nagao et al. 2006; Dinakar and Bartels 2013). Our results indicated that the gametophores of *P. replicatum* accumulated soluble sugars in response to 10 µM ABA treatment and in response to moderate osmotic stress (400 mM sorbitol) and salt stress (200 mM NaCl) (Fig. 5). No alterations in the QY values were observed under such conditions (data not shown). Previous reports on vascular and nonvascular plants have indicated that application of exogenous ABA induces accumulation of soluble sugars (Orr et al. 1986; Bravo et al. 1998; Bhyan et al. 2012; Mayaba et al. 2001; Nagao et al. 2005, 2006; Akter et al. 2014; Takezawa et al. 2015). Interestingly, in *P. replicatum*, application of exogenous ABA induced accumulation of soluble sugars to an extent comparable to that induced by NaCl. This finding could suggest that NaCl-induced accumulation of soluble sugars is mediated by ABA in *P. replicatum*. Moderate osmotic stress also induced sugar accumulation, possibly via an endogenous ABA-dependent pathway. Another possible explanation is that both NaCl and osmotic stress activated similar response pathways in an ABA-independent manner. Whether *P. replicatum* changes its internal ABA content in response to NaCl or osmotic stress is a main question that we will address in the near future. Finally, we speculate that the observed decrease in the rate of water loss during dehydration (Fig. 3a) and the further increase in the velocity of QY recovery after rehydration (Fig. 3b) may be due, at least in part, to the ABA-mediated accumulation of soluble sugars in the gametophores of *P. replicatum*.

### ABA affects spore germination and protonemal development in *P. replicatum*

ABA is a phytohormone that participates in plant development and in responses to different biotic and abiotic stresses (Humplík et al. 2017; Ma et al. 2018). To address the role of ABA during the early *P. replicatum* developmental stages, the effects of ABA on spore germination and protonemal growth were analysed. Spore germination of *P. replicatum* was less sensitive to ABA than seed germination of vascular plants (Fig. 6a). In Arabidopsis, it is well known that low ABA concentrations ranging from 3 to 5 µM are enough to completely inhibit seed germination (Finkelstein 1994; Arenas-Huertero et al. 2000). However, up to 50 µM ABA was required to inhibit 50 % of the spore germination of *P. replicatum* (Fig. 6a). Two important observations contradicted previous findings regarding *P. patens* spore germination (Vesty et al. 2016). The sensitivity of *P. patens* spores to ABA seems to be higher than that of *P. replicatum* spores because only 2 µM ABA is sufficient to significantly delay *P. patens* spore germination. In this study, we found that *P. replicatum* exhibited a better germination rate than *P. patens*. On the other hand, our protonema assay revealed that after 7 d of ABA treatment, the protonemata showed a phenotype characterized by cell greening, decreased cell length, and a round shape, consistent with the brood cell phenotype commonly observed to develop in *P. patens* in response to ABA (Fig. 6b, c) (Decker et al. 2006; Takezawa et al. 2011; Stevenson et al. 2016; Shinozawa et al. 2019; Arif et al. 2019); these results indicate that *P. replicatum* apical cells retain polar growth under such conditions. These findings support early ABA participation in bryophytes during land conquest and vegetative dispersion since brood cells function as dormant structures.

### *P. replicatum* transcriptome changes in response to dehydration

Recently, *de novo* transcriptome assembly has been performed for gametophores of DT mosses, such as *S. caninervis* (Gao et al. 2014) and *B. argenteum* (Gao et al. 2015, 2017), under conditions of dehydration. In all three reports gametophores were tested for dehydration, and further RNA-Seq strategies were performed. However, no work has used RNA-Seq to analyse the protonemata of a DT moss in response to dehydration. To preliminarily investigate the transcriptomic response to a low-water regime in *P. replicatum* protonemata, we assessed the effects of fast dehydration of protonemal tissues at 23% RH over short time periods: 10 min (T1) and 30 min (T2). The RNA-Seq analyses revealed that compared with genes in the control condition, 1368 genes at T1 and 4220 genes at T2 were differentially expressed. Bioinformatics analysis of the DEGs reduced the numbers to 972 upregulated and 821 downregulated DEGs. Of these, a total of 175 DEGs that were homologous with *P. patens* sequences were significant when both treatments were considered together (T1 + T2), and the most abundant were upregulated genes. Consistent with the scenario in *P. replicatum*, the mosses *S. caninervis* and *B. argenteum* show an inducible molecular response to dehydration. However, the inducible molecular mechanism used by *P. replicatum* is completely different from that used by the moss *T. ruralis*, another FDT plant that uses a constitutive mechanism to survive desiccation (Oliver et al. 2000) but uses an inducible mechanism during rehydration (Oliver et al. 2005). This mechanism also seems to be partially present in *B. argenteum* (Gao et al. 2017). It could be interesting to investigate the molecular response of *P. replicatum* during the process of rehydration; future studies will address this topic. Nuclear and chloroplastic protein-encoding genes were the most abundant types of genes among the induced genes, accounting for more than 50% of the genes; in contrast, nuclear protein-encoding genes were the most abundant proteins among the repressed genes (31.8%). Downregulated transcripts encoding chloroplastic proteins accounted for 18.2% of the genes; among them, genes encoding important proteins required for assembly of the PSII reaction centre, such as PsbN (Torabi et al. 2014), several photosynthetic electron transfer B proteins (Goltsev et al. 2021), and PHY1 light-sensor protein kinase (Possart and Hiltbrunner 2013), were identified. Notably, inhibition of photosynthesis is a primary central response that is exhibited by both desiccation-tolerant and desiccation-sensitive plants affected by drought stress to protect the photosynthetic apparatus (Challabathula et al. 2018).

Desiccation-tolerant plants employ both conserved and novel antioxidant enzymes/metabolites to minimize oxidative damage and to protect the photosynthetic machinery. *De novo*-synthesized stress-induced proteins, in combination with antioxidants, are important components of the protective network. Interestingly, transcripts encoding proteins associated with photosynthesis (such as thiamine thiazole synthase; the early light inducible protein ELIP1; light-harvesting PSII subunits; the MPH1 protein, which is involved in Maintenance of PSII under High light conditions; ribulose biphosphate carboxylase small chain; chlorophyll a-/b-binding proteins; plastocyanin; and PSI-F), general/sugar metabolism (enolase, fructose-bisphosphate aldolase, D-fructose-1,6-bisphosphate-1-phosphohydrolase, phosphopyruvate hydratase, phosphoglycerate kinase, malate dehydrogenase, transketolase, and xyloglucan endotransglucosylase/hydrolase), and translation (the 60 S acidic ribosomal protein P0; the 40 S ribosomal proteins S3a, S25, and S21; peptidyl-prolyl alpha-trans isomerase; 5-methyltetrahydropteroyltriglutamate-homocysteine S-methyltransferase; the 60 S ribosomal protein L7a; and elongation factor 1-alpha), among other processes were found upregulated. Thus far, studies have revealed that most of these genes participate in responses to abiotic stress in plants either by expression analysis or forward and reverse genetics Bernacchia et al. 1995; Laxalt et al. 1996; Haldrup et al. 2000; Sharma et al. 2003; Morita-Yamamuro et al. 2004; Barkla et al. 2009; Lu et al. 2012; Omidbakhshfard et al. 2012; Hertz et al. 2013; Wang et al. 2013; Gururani et al. 2015; Liu et al. 2015; Liu and Last 2015; Piro et al. 2015; Cai et al. 2016; Joshi et al. 2016; Li et al. 2016; Yasmenn et al. 2016; Wang et al. 2016; Giarola et al. 2017; Zhou et al. 2018; Li et al. 2019; Hasanuzzaman et al. 2020; Zhao et al. 2020). During abiotic stress, proteins can be damaged, and turnover of specific proteins is required. Thus, upregulation of transcripts encoding proteins related to translation could be a strategy of *P. replicatum* to adapt to dehydration.

On the other hand, transcripts related to osmoprotection, oxidative stress, protein integrity/turnover (protection, repair, degradation), and signalling also accumulated during drying but were more closely related to Arabidopsis, wheat, and other plant species than *P. patens* (germin-like proteins, several LEA proteins, the early response to dehydration protein ERD15, aquaporins, cobalamin synthase, cytochrome c oxidase, an HR-like lesion-inducing protein-related protein, plasma membrane H+-ATPases, ubiquitin, polyubiquitin, the glycine cleavage system P protein, alpha/beta-hydrolases, Shaggy-like kinase 13, serine/threonine kinases, and basic region leucine zipper transcription factors, among others).

Finally, using the PlantCARE database, we evaluated the promoter sequences of all upregulated and downregulated genes. We found 27 promoters enriched with ABRE and DRE boxes, which are cis-elements that bind to transcription factors in response to ABA and abiotic stress, respectively (Guiltinan et al. 1990; Yamaguchi-Shinozaki and Shinozaki 1994). The ABRE sequence was the most abundant motif in both induced and repressed genes, supporting the idea of an important role for ABA during the *P. replicatum* dehydration response. The upregulated genes whose promoters were enriched mainly with ABRE boxes encoded proteins involved in the photosynthetic process. These observations support the idea that dehydration induces a rapid change in the expression profile of *P. replicatum* to readjust metabolism so that the organism can respond efficiently to stress. Further analyses of the molecular responses of *P. replicatum* during different developmental stages and under other conditions of dehydration will help elucidate the molecular strategy of this DT moss. Nevertheless, this study represents the first RNA-Seq study on protonemal tissues of an FDT moss. Altogether, our results indicate that *P. replicatum* is an FDT moss that exhibits an inducible mechanism in response to abiotic stress and ABA. This work provides a basis for ongoing studies on the functional genomics of *P. replicatum* as a plant model for the study of DT.

## Electronic Supplementary Material

Below is the link to the electronic supplementary material.

Supplementary file1 (DOC 872 kb)

Supplementary file1 (XLSX 20 kb)

Supplementary file1 (XLSX 81 kb)
