# Supplementary material for: Pseudocrossidium replicatum (Taylor) R.H. Zander is a fully desiccation-tolerant moss that expresses an inducible molecular mechanism in response to severe abiotic stress
Source: Plant Mol Biol. 2021 Jun 29;107(4-5):387–404. doi: 10.1007/s11103-021-01167-3 (PMC8648698; doi:10.1007/s11103-021-01167-3)
Supplement: Supplementary file 1 — Supplementary file1 (DOC 872 kb) [file 11103_2021_1167_MOESM1_ESM.docx]

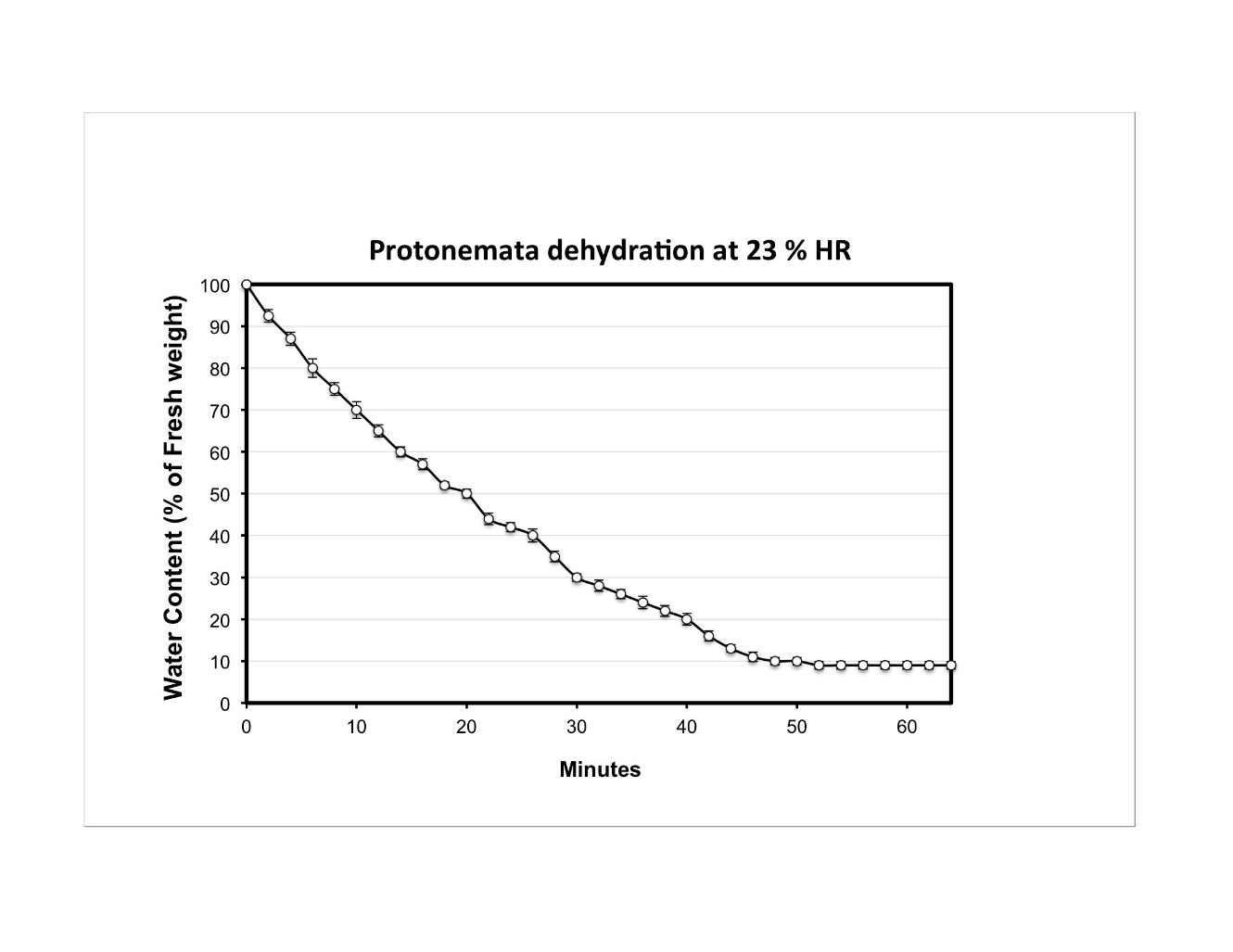


Fig. S1a**.**  Dehydration kinetics of *P. replicatum* 7-d-old protonema exposed to 23% RH atmosphere.


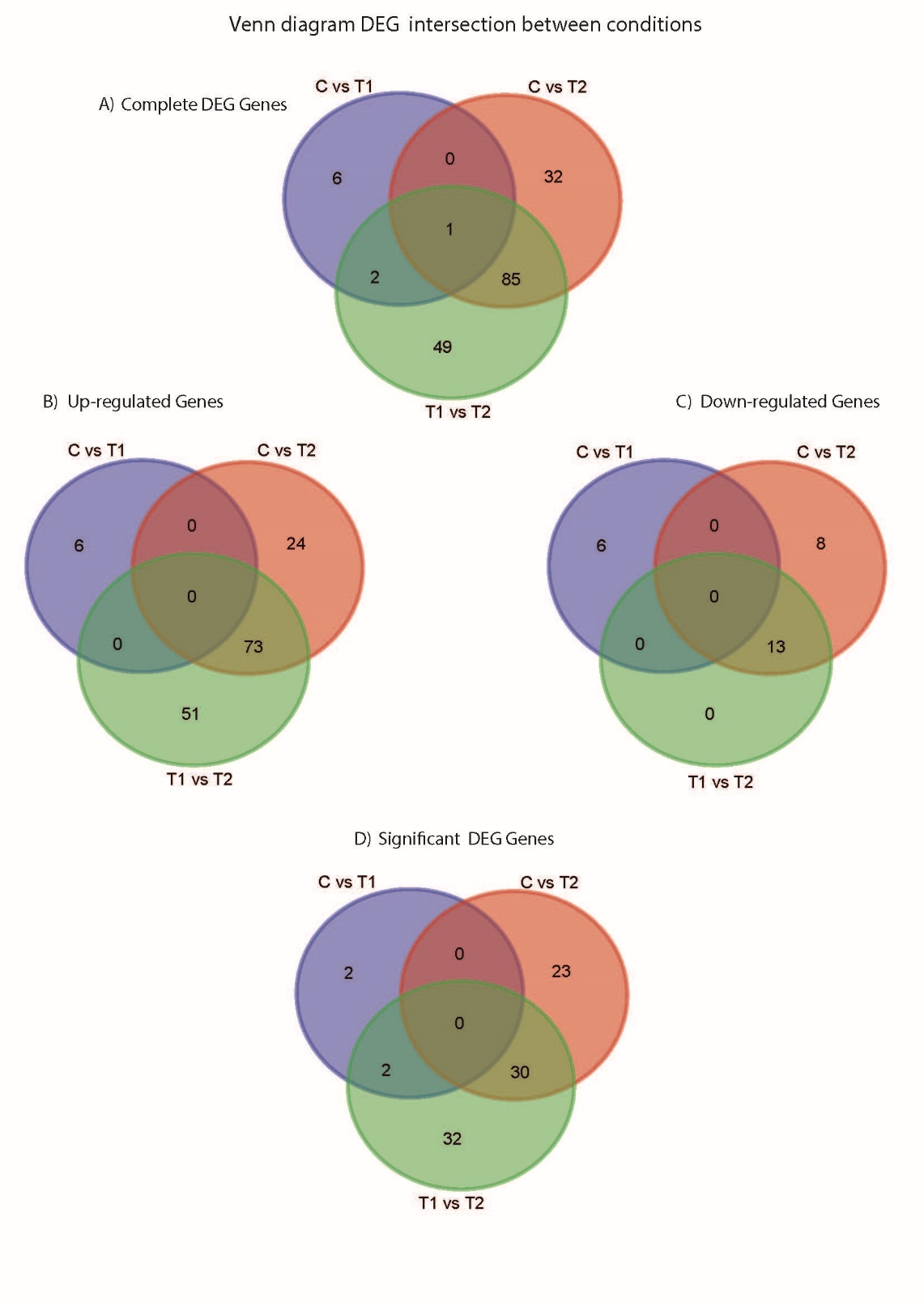


Fig. S1b Venn diagram after the statistical analysis of the RNA-Seq results.  A) Total number of global genes, B) Up-regulated genes, C) Down-regulated genes and D) Significant mapped to *P. patens*.


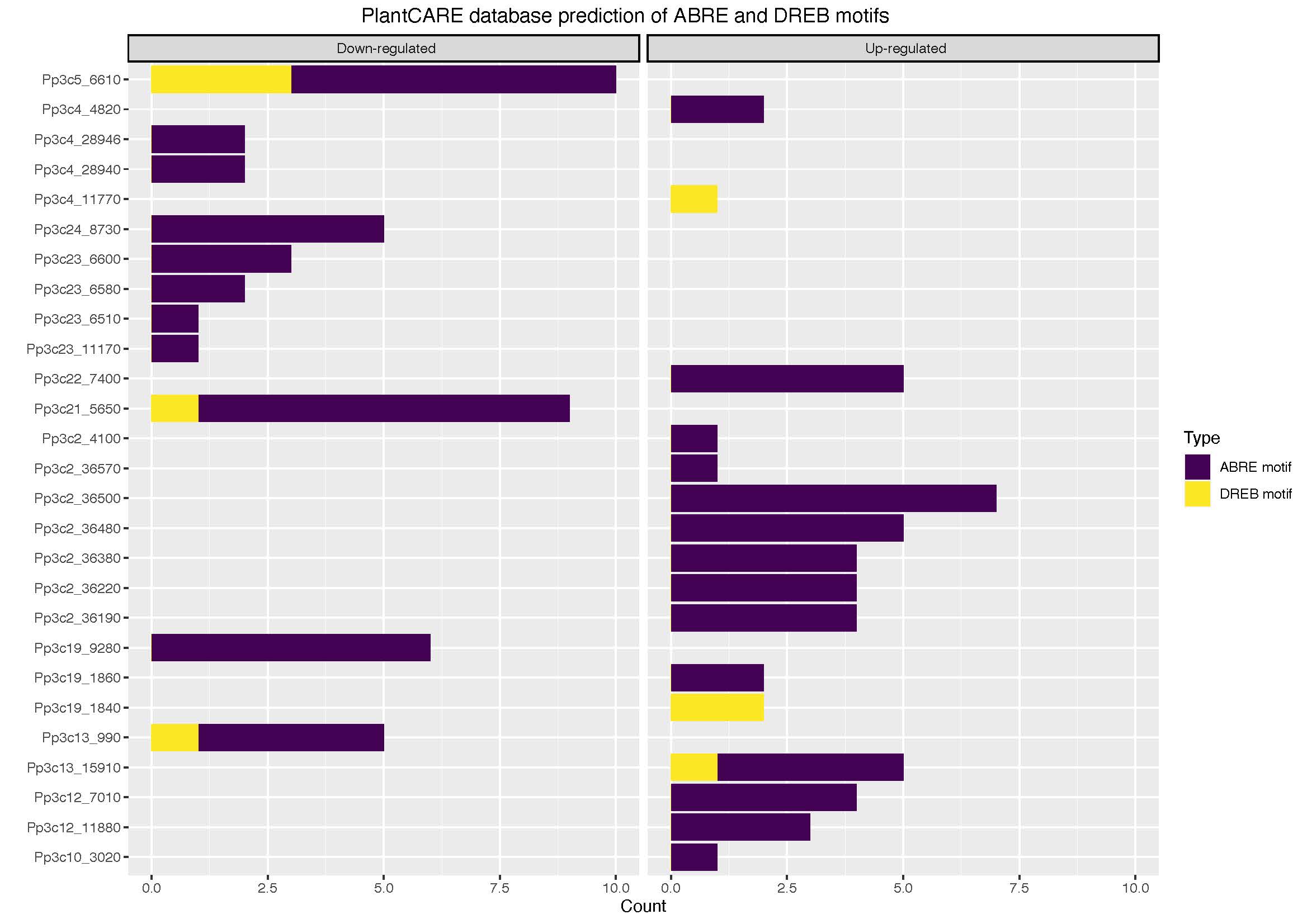


Fig. S1c PlantCARE database prediction of ABRE and DREB *cis*-acting regulatory sequences into the 2 Kb nucleotide upstream of the ATG of the up-regulated and down-regulated genes obtained in the RNA-Seq analysis. The analysed sequences correspond to the *P. patens* homologue genes. The table shows the numbers of ABRE and DREB motifs predicted for each promoter region.


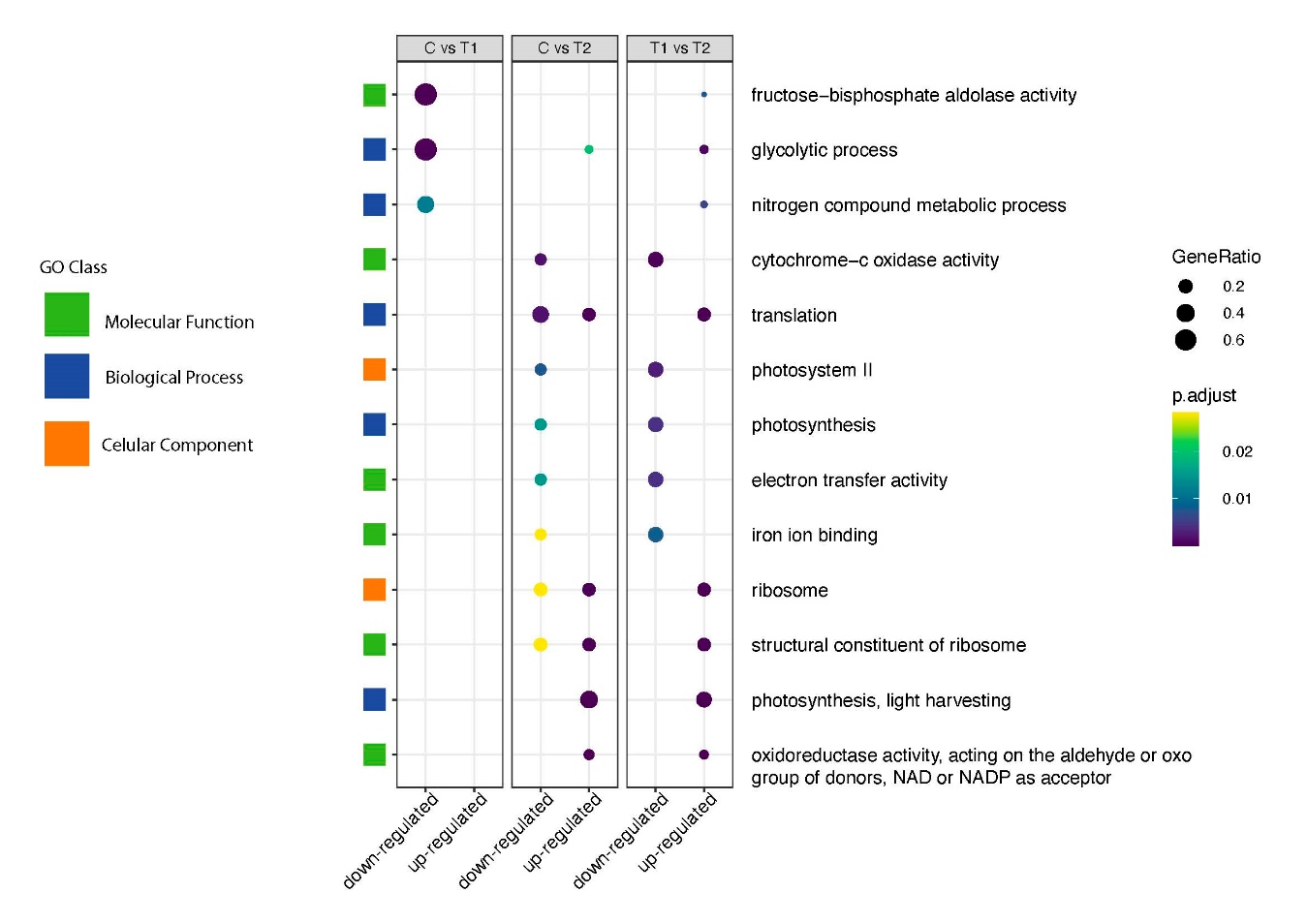


Fig. S1d GO Enrichment analysis was performed using UNIPROT. *P. patens* annotation with enrich function in R (Clusterprofiler) was used to compare and plot the up-regulated and down-regulated genes based on a GO standardized DAG structured vocabulary system.


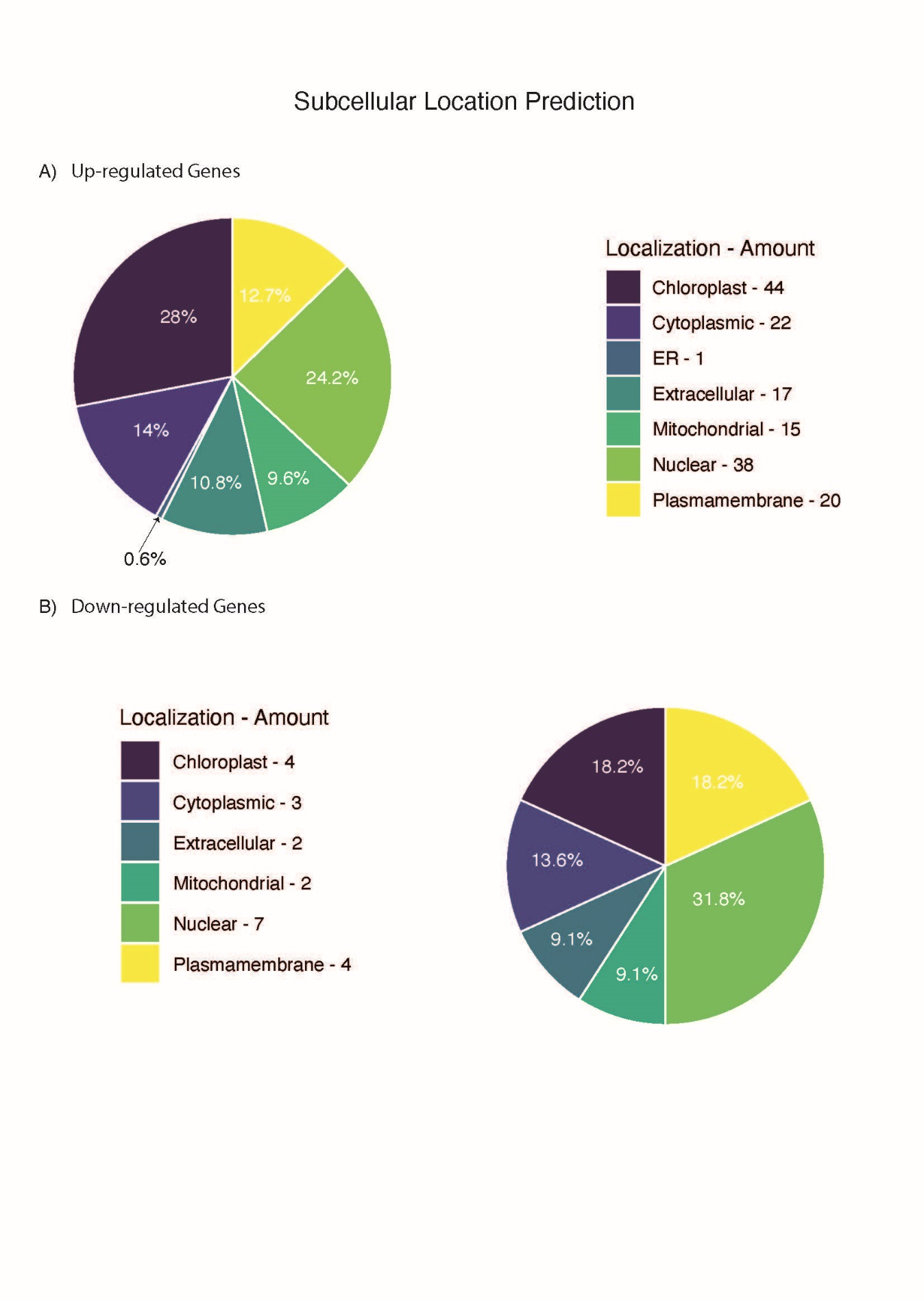


Fig. S1e Predicted subcellular localization (CELLO2GO) of proteins encoded by up-regulated (A) and down-regulated (B) genes, respectively.
